# High-Resolution Array CGH Profiling Identifies Na/K Transporting ATPase Interacting 2 (NKAIN2) as a Predisposing Candidate Gene in Neuroblastoma

**DOI:** 10.1371/journal.pone.0078481

**Published:** 2013-10-25

**Authors:** Paolo Romania, Aurora Castellano, Cecilia Surace, Arianna Citti, Maria Antonietta De Ioris, Pietro Sirleto, Marilena De Mariano, Luca Longo, Renata Boldrini, Adriano Angioni, Franco Locatelli, Doriana Fruci

**Affiliations:** 1 Paediatric Haematology/Oncology Department, Bambino Gesù Children's Hospital, IRCCS, Rome, Italy; 2 Cytogenetics and Molecular Genetics Unit, Bambino Gesù Children's Hospital, IRCCS, Rome, Italy; 3 Pathology Department, Bambino Gesù Children's Hospital, IRCCS, Rome, Italy; 4 Immunological Therapy, IRCCS A.O.U. San Martino-IST, Istituto Nazionale per la Ricerca sul Cancro, Genoa, Italy; 5 Pediatrics Department, University of Pavia, Pavia, Italy; Sanjay Gandhi Medical Institute, India

## Abstract

Neuroblastoma (NB), the most common solid cancer in early childhood, usually occurs sporadically but also its familial occurance is known in 1-2% of NB patients. Germline mutations in the *ALK* and *PHOX2B* genes have been found in a subset of familial NBs. However, because some individuals harbouring mutations in these genes do not develop this tumor, additional genetic alterations appear to be required for NB pathogenesis. Herein, we studied an Italian family with three NB patients, two siblings and a first cousin, carrying an *ALK* germline-activating mutation R1192P, that was inherited from their unaffected mothers and with no mutations in the *PHOX2B* gene. A comparison between somatic and germline DNA copy number changes in the two affected siblings by a high resolution array-based Comparative Genomic Hybridization (CGH) analysis revealed a germline gain at *NKAIN2* (Na/K transporting ATPase interacting 2) locus in one of the sibling, that was inherited from the parent who does not carry the ALK mutation. Surprisingly, NKAIN2 was expressed at high levels also in the affected sibling that lacks the genomic gain at this locus, clearly suggesting the existance of other regulatory mechanisms. High levels of NKAIN2 were detected in the MYCN-amplified NB cell lines and in the most aggressive NB lesions as well as in the peripheral blood of a large cohort of NB patients. Consistent with a role of NKAIN2 in NB development, NKAIN2 was down-regulated during all-trans retinoic acid differentiation in two NB cell lines. Taken together, these data indicate a potential role of NKAIN2 gene in NB growth and differentiation.

## Introduction

Neuroblastoma (NB), the most common extracranial solid tumor of childhood, originates from sympathetic neuronal progenitor cells and accounts for more than 15% of all pediatric cancer deaths [1], [2]. The heterogenous clinical behaviour, ranging from localized mass that undergo spontaneous regression, to widely disseminated NB with a survival rate of 50% despite intensive multimodal cytotoxic therapy, is attributable to both biological and genetic characteristics of the tumor [3].

Somatically acquired genomic aberrations are of fundamental importance for predicting NB patients' outcome. Tumors with amplification and overexpression of the *MYCN* proto-oncogene or allelic loss of chromosome arms 1p and 11q or both, typically are metastatic at diagnosis and therapy-resistant [1]. Conversely, tumors showing hyper- or hypoploidy due to whole-chromosomal gains or losses, respectively, are more easily cured and frequently undergo spontaneous regression [1].

Approximately 1–2% of NB cases have a NB-positive familial history with patients either distributed along three generations or clustered in the most recent one [1]. Mutations in *paired-like homeobox 2B* (*PHOX2B*) and the tyrosine kinase receptor *anaplastic lymphoma kinase* (*ALK*), two genes predominantly expressed in the developing peripheral nervous system, have been identified as predisposing events for NB. Germline missense mutations in *PHOX2B* were originally observed in a familial case of NB and in a patient with NB associated with congenital central hypoventilation syndrome (CCHS) and/or Hirschsprung disease [4]. Subsequent studies confirmed the association of *PHOX2B* mutations with NB predisposition [5]–[7], and showed that *PHOX2B* mutations could also be observed as *de novo* mutations in apparently sporadic tumors [8], [9]. More recently, *ALK* has been demostrated to be constitutively activated by gene mutations in the tyrosine kinase domain and/or amplification in sporadic [10]–[13], as well as in familial cases of NB [14], [15]. The range of somatic and germline *ALK* mutations are different, being some mutations detected either in familial or in sporadic forms of NB.

Although germline mutations of *ALK* and *PHOX2B* account for the majority of familial NB, additional genetic factors responsible for NB tumor progression may still be discovered. Consistently, a recent paper demonstrates that *ALK* deleterious mutations are rare events in NB patients with a high probability of predisposition [16], strongly suggesting that other predisposing genes remain to be identified.

Recently, the application of whole genome high-resolution array comparative genomic hybridization (array CGH) techniques has allowed the identification of small copy number changes in cancer genomes [17]. Taking advantage of this technology, we screened NB-affected and unaffected individuals of an Italian family to systematically identify germline and tumor-specific copy number alterations relevant to NB. We identified 3 new genomic alterations in chromosomal regions devoid of any copy number variations (CNV). One of these genes, *NKAIN2*, was detected upregulated in a large cohort of NB patients (both tumor and blood speciments) and NB cell lines with aggressive phenotype, suggesting its putative role in NB pathogenesis.

## Results

### Neuroblastoma family case

**Figure 1:**
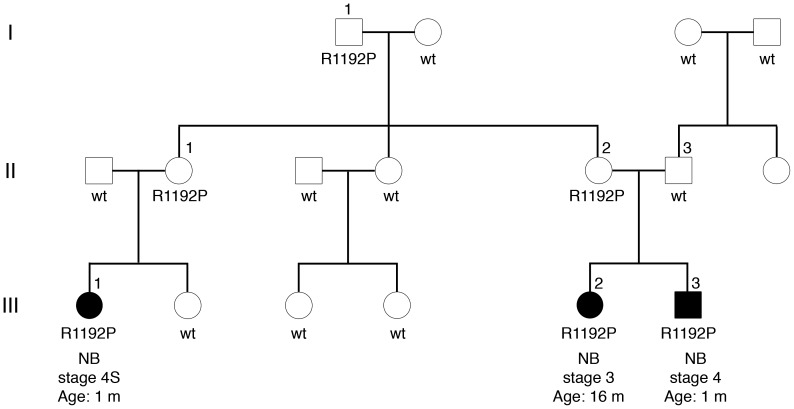
Pedigree of a novel Italian NB family with *ALK* mutations. Family includes three affected children, two siblings and a first cousin indicated by filled symbol. Patient III-1 has an adrenal NB at stage 4S of disease. Patient III-2 was affected by a stage 3 NB in abdominal paravertebral region. Patient III-3 had a stage 4 adrenal NB with multiple subcutaneous lesions. All family members with DNA available for genotyping are indicated with either wild type (wt) for *ALK*, or with mutation in the *ALK* tyrosine kinase domain (R1192P). Stage of disease and morphologic tumor classification are listed according to the INSS and the INPC, respectively. Age: age at diagnosis; m: months; wt: wild-type in the *ALK* gene, R1192P: R1192P mutation in the *ALK* gene.

This family includes three NB patients, two siblings and a first cousin (Figure 1). The two siblings were affected by stage 3 and stage 4 NB according to INSS [18], (III-2 and III-3, respectively, Figure 1). The sibling III-2 is the eldest daughter of non-consanguineous parents. She was diagnosed at age of 16 months with an abdominal paravertebral *MYCN*-non amplified and 1p deletion negative NB and underwent chemotherapy according to the European unresectable NB protocol [19] before tumor surgical resection with minimal residual disease. Her younger brother, i.e. III-3, was diagnosed at age of 20 days with a *MYCN* gain and 1p deletion positive adrenal NB with multiple subcutaneous nodules and bone metastasis. He received chemotherapy according to the SIOP European NB Infant protocol trial 99.3 before underwent surgery on primary tumor with minimal residual disease. The first cousin III-1 had a stage 4S adrenal NB and laterocervical lymphonodes diagnosed at the age of 1 month. She underwent chemotherapy, surgery and radiotherapy on primary tumor bed [20]. All patients had good prognosis: III-1 and III-3 are currently in complete remission and III-2 is in minimal residual disease at 24 6/12 years, 3 10/12 years, 3 5/12 years from diagnosis, respectively.

### Mutational screening of *PHOX2B* and *ALK* genes

Direct DNA sequencing analysis of *PHOX2B* and *ALK* genes was performed on the constitutional DNA from blood specimens of the three NB patients (Figure 1). No mutation was found in the three exons of *PHOX2B* (data not shown). Conversely, DNA sequences of exons 20 to 29 encoding the tyrosine kinase domain of *ALK* revealed a guanosine-to-cytosine change in exon 23, resulting in an arginine-to-proline substitution at codon 1192 (R1192P) in all three NB patients (Figure 1). DNA sequencing of their relatives indicated that the R1192P mutation was inherited from the unaffected mothers and the grandfather (II-1, II-2 and I-1, respectively; Figure 1). Since this *ALK* mutation was inherited from asymptomatic parents, additional genetic events are thought to be required for tumor development in these patients.

### Array CGH analysis of somatic and germline DNA copy number changes in NB-affected siblings

**Figure 2:**
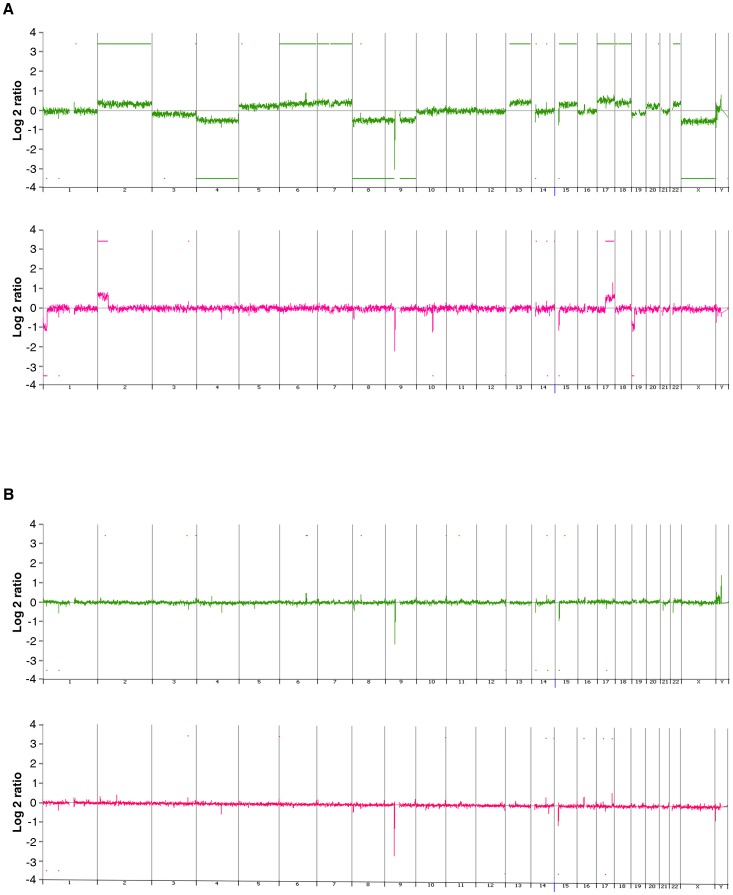
Array CGH copy number profiles of tumor and constitutional DNA samples from the affected siblings. Array CGH results from tumor (A) and constitutional (B) DNA analysis of the affected siblings (patient III-2 and III-3, in green and red, respectively). The X-axis represents the chromosomes, while the Y-axis represents the normalized log2 Cy5(patient)/Cy3(healthy control) fluorescence intensity thresholds -1 (loss) and 1 (gain), respectively. Array CGH results from tumor DNAs (A) revealed gains and losses of whole chromosome in stage 3 NB (patient III-2, in green) and partial chromosome imbalances in stage 4 NB (patient III-3, in red). Array CGH results from constitutional DNAs (B) revealed gains and losses of small chromosomal regions.

**Figure 3:**
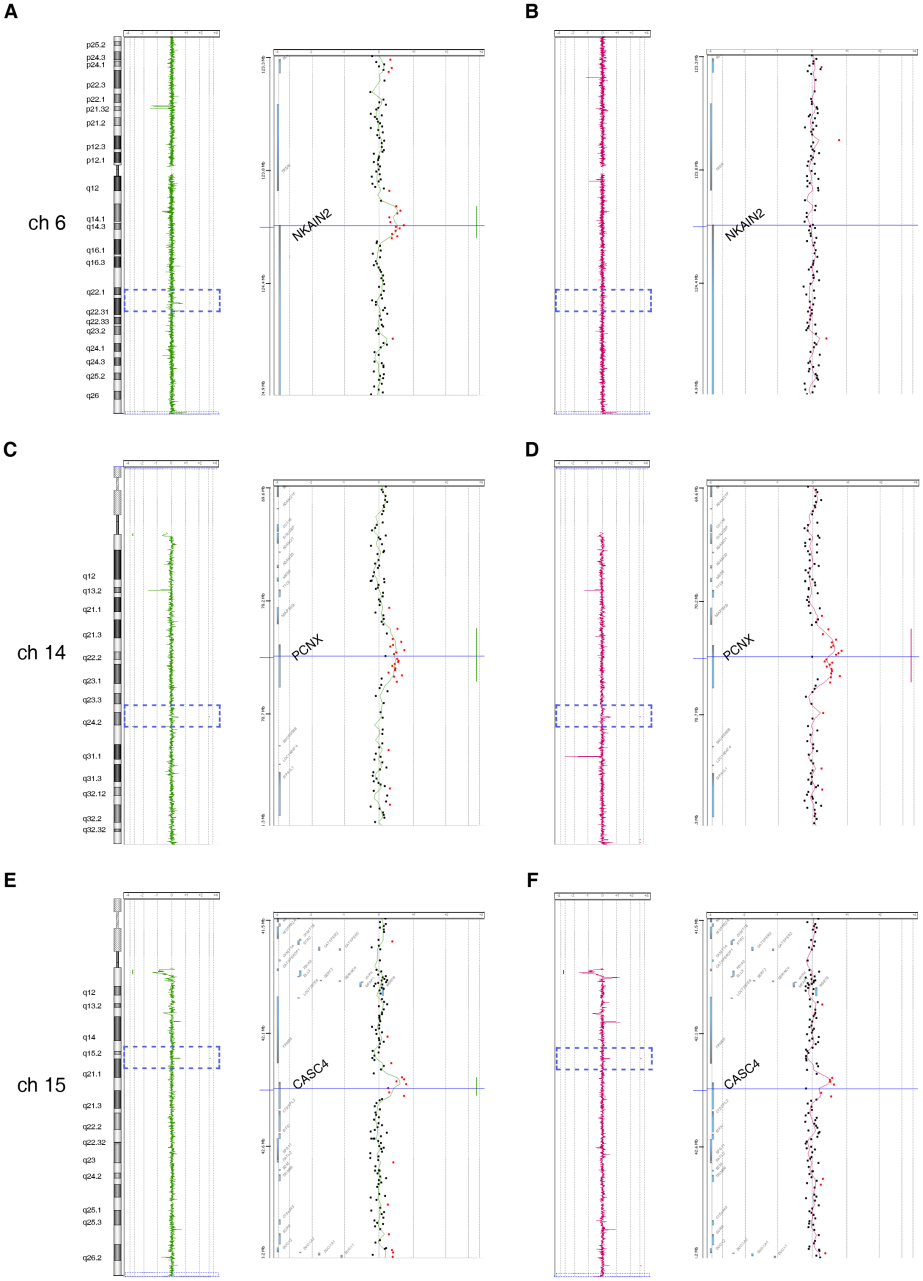
Copy number alterations of chromosomes 6, 14 and 15 in the affected siblings. The chromosome 6, 14 and 15 views (left) display the copy number profile of analysing constitutional DNA of III-2 (A, C and E) and III-3 (B, D and F) patients versus normal references. The gene views (right) magnify the selected amplification regions at 6q22, 14q24 and 15q15 indicating the log2 copy number ratios of individual oligos.

To search for potential common chromosomal alterations indicating a putative predisposing NB locus, an array CGH analysis was performed on tumor and constitutional (peripheral blood) DNA of the affected siblings III-2 and III-3 (Figure 2). Array CGH analysis of tumor DNA revealed whole chromosome losses or gains in III-2, and partial chromosome imbalances in III-3 (Figure 2A, III-2 and III-3 depicted with green and red, respectively). In III-2, eight chromosomes (2, 6, 7, 13, 15, 17, 18 and 22) were entirely duplicated and four chromosomes (4, 8, 9 and X) were completely lost (Figure 2A). In III-3, the most prominent copy number alterations affected the short arm of chromosomes 1, 2 and 19 and the long arm of chromosomes 10, 14 and 17, where distinct gains and losses including loci for several genes were detected (Figure 2A). However, no remarkable alterations could orientate to any particular predisposing locus. When array CGH analyses of the DNA tumor samples (Figure 2A) were compared with matched constitutional DNA (Figure 2B), three genomic gains were identified in regions devoid of copy number variations (CNVs) at chromosomes 6q22, 14q24 and 15q15 that include loci for *NKAIN2* (Na/K transporting ATPase interacting 2), *PCNX* (pecanex homolog) and *CASC4* (cancer susceptibility candidate 4), respectively (Figure 3 and Table 1). These genomic gains are depicted in figures 3A, 3C and 3E for III-2 and figures 3B, 3D and 3F for III-3. The genomic gain at *NKAIN2* was detected in sibling III-2 (Figure 3A) but not in sibling III-3, while the gains at *PCNX* and *CASC4* were found in both siblings (Figure 3C-F). To see whether these genomic gains were shared with the affected first cousin III-1, array CGH analysis was performed similarly on the constitutional DNA sample. In this NB patient, none of these genomic gains were detected (Figure S1). Next, to define which parent transmits these genomic alterations to their offspring, array CGH analysis was performed also on the constitutional DNA of the parents (i.e., mother II-2 and father II-3, respectively) of the affected siblings. The genomic gains in all *NKAIN2*, *PCNX* and *CASC4* loci were found in II-3 (Figure S2) who does not carry the R1192P mutation of *ALK*, but not in II-2 who does carry the R1192 mutation.

**Table 1:** Array-CGH results in tumor and peripheral blood DNA samples.

	Tumor DNA			Constitutional DNA		
Cases	aCGH	Size (kb)	Genes	aCGH	Size (kb)	Genes
III-2	dup(6q22.31)	333		dup(6q22.31)	333	no genes
	dup(6q22.31)	156		dup(6q22.31)	156	***NKAIN2***
	dup(14q24.2)	258		dup(14q24.2)	258	***PCNX***
	dup(15q15.3)	90		dup(15q15.3)	90	***CASC4***
III-3	del(1p36.33)	19		dup(14q24.2)	258	***PCNX***
	dup(2p25.3)	48		dup(15q15.3)	90	***CASC4***
	del(10q22)	3				
	dup(14q24.2)	0.26				
	dup(17q21.2)	41				
	del(19p13.3)	14				
III-1				del(2p16.3)	132	no genes
				del(3p22.1)	145	*ULK4*
II-2				dup(1p36.23)	37	*VAMP3*
				dup(1p36.21)	24	*PER3*
						*VPS13D*
II-3				dup(6q22.31)	333	no genes
				dup(6q22.31)	156	***NKAIN2***
				dup(14q24.2)	258	***PCNX***
II-1				del(2p16.3)	132	no genes
				dup(6q27)	453	no genes

### *NKAIN2* is expressed at high levels in the affected siblings

**Figure 4:**
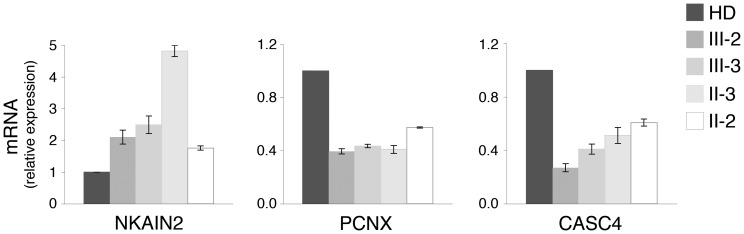
Expression of *NKAIN2, PCNX* and *CASC4* in the affected siblings and their parents. qPCR analysis of mRNAs from age-matched healthy donors (HD, n = 6), the affected sibling (III-2 and III-3) and their parents (II-2 and II-3). β-actin was used for normalization. The error bars represent the mean ± SD (n = 3).

To determine whether genomic gains detected by array CGH resulted in enhanced gene expression *NKAIN2*, *PCNX* and *CASC4* mRNA levels were evaluated in the affected siblings and their parents by qPCR. High *NKAIN2* expression (P<0.01) was found in both individuals (II-3 and III-2) who carry the genomic gain at *NKAIN2* as compared to healthy donors (Figure 4). Surprisingly *NKAIN2* was expressed at high levels also in the sibling III-3 who lacks the genomic gain, suggesting the occurance of additional mechanisms for *NKAIN2* gene regulation (Figure 4). Conversely, genomic gains at *PCNX* and *CASC4* did not result in enhanced gene expression, but rather in a reduction. Hence, the latter two genes were not further investigated.

### *NKAIN2* is downregulated during neuronal differentiation of NB cell lines

**Figure 5:**
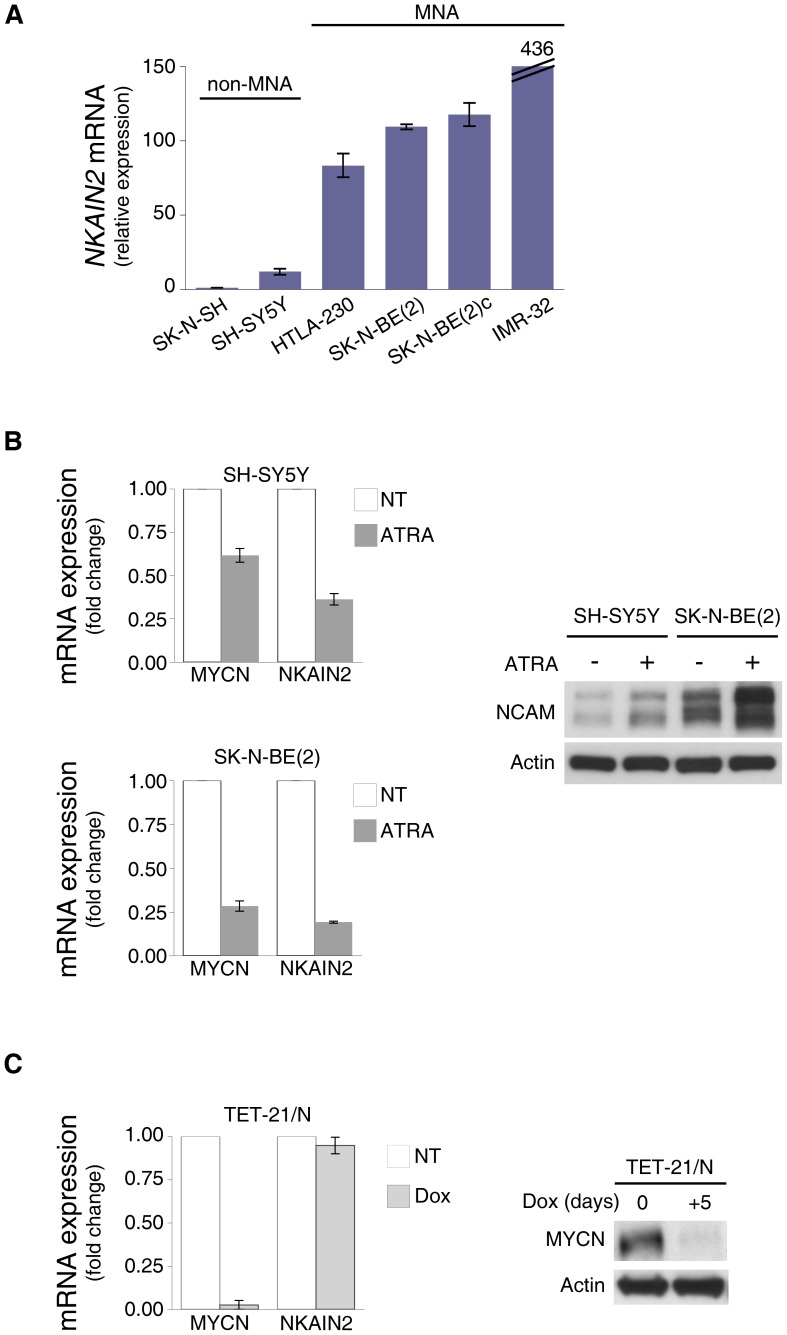
Expression of *NKAIN2* in NB cell lines can be reduced by ATRA treatment. A, qPCR analysis of mRNAs from different NB cell lines with or without MYCN amplification (MNA). β-actin was used for normalization. B, qPCR analysis of MYCN and NKAIN2 mRNA expression (left panel) and immunoblot analysis of NCAM expression (right panel) in SH-SY5Y and SK-N-BE(2) cells untreated or treated with ATRA for 4 and 10 days, respectively. C, qPCR analysis of MYCN and NKAIN2 expression (left panel) and immunoblot analysis of MYCN expression (right panel) in Tet-21/N cells untreated or treated with doxycycline (Dox) for 5 days. The error bars represent the mean ± SD (n = 3).

To investigate how *NKAIN2* expression may contribute to tumor initiation and/or clinical phenotype, we next evaluated NKAIN2 mRNA levels on several NB cell lines without copy number changes at 6p [21]–[23], in order to avoid the influence of somatic DNA alterations on gene expression. The qPCR revealed that *NKAIN2* was expressed at higher levels in the MYCN-amplified NB cell lines [24], as compared to the less aggressive non-MYCN-amplified NB cell lines (Figure 5A).

To determine whether NKAIN2 is involved in NB cell differentiation, two ATRA-sensitive NB cell lines, SH-SY5Y and SK-N-BE(2), were daily treated with ATRA for 4 and 10 days respectively, resulting in distinct morphological changes with extensive neurite outgrowth. Differentiation was also confirmed through the observed down-regulation of MYCN and upregulation of NCAM at mRNA and protein levels, respectively (Figure 5B). As shown in Figure 5B, ATRA treatment induced detectable downregulation of NKAIN2 expression in both SH-SY5Y and SK-N-BE(2) cell lines, suggesting a potential involvement of this gene in NB growth and differentiation.

The coordinated expression of NKAIN2 and MYCN indicates the possibility that NKAIN2 is regulated by MYCN. To address the regulatory role of MYCN in the NKAIN2 expression, we utilized a MYCN-transfected NB cell line, Tet-21/N, carrying a tetracycline-repressible MYCN transgene [25]. Tet-21/N cells untreated or treated with doxycycline, were tested for the expression of MYCN and NKAIN2. MYCN expression was drastically suppressed by doxycycline treatment in Tet-21/N, as determined by qPCR and Western blotting (Figure 5C). However, this clear change of MYCN expression, did not induce a proportional change in the expression of NKAIN2 (Figure 5C), suggesting that MYCN is not directly involved in the regulation of NKAIN2 expression in NB cell lines.

### Expression of NKAIN2 is significantly higher in the most aggressive NB lesions and the constitutional DNA of NB patients

**Figure 6:**
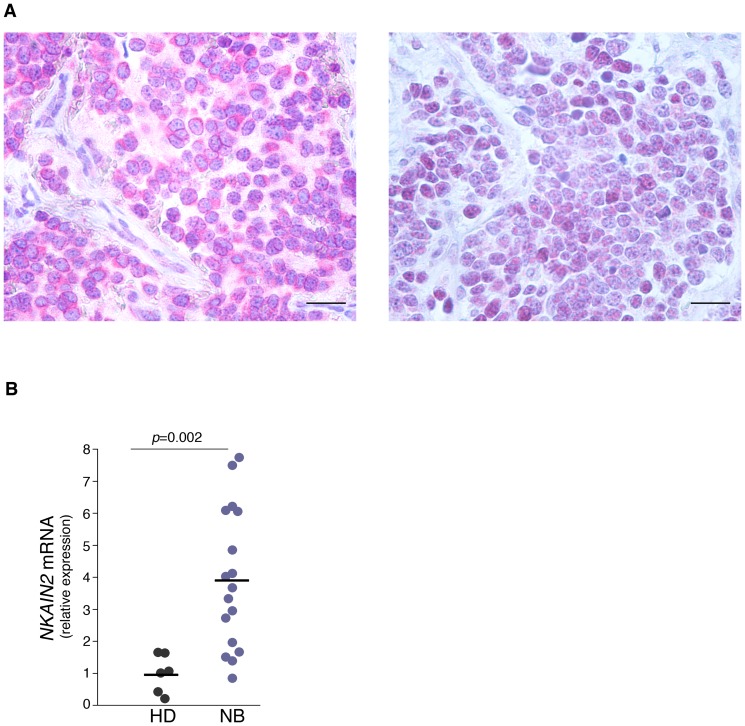
Expression of *NKAIN2* in primary NB lesions and peripheral blood of NB patients. A, Immunohistochemistry of human NB tissues section with Ab to NKAIN2. Visualized with Fast Red (red), nuclei counter-stained with haematossilin (blue). NKAIN2 is strongly expressed on the cell membrane of neuroblasts in the high-risk NB (left panel) and undetectable in the neuroblasts of low-risk NB (right panel). Original magnification, ×40. Scale bars 30 µm. Data shown are representative of 7 high-risk and 5 low-risk NB. B, qPCR analysis of mRNA in 6 healthy donors (HD) and 17 NB patients in remission for at least 1 year (median age  = 6 for both HD and NB patients). β-actin was used for normalization. Significant differences between HD and NB patients were evaluated by Mann-Whitney test.

Given the potential role of NKAIN2 in NB differentiation, expression of NKAIN2 was investigated in 12 primary NB lesions, including 5 low-risk and 7 high-risk NB, by immunohistochemistry. NKAIN2 was clearly detected on the cell membrane and cytoplasm of neuroblastic cells in 7/7 high-risk NB. Conversely, NKAIN2 was hardly seen in the neuroblastic cells of 5/5 low-risk NB. A representative staining revealing expression levels and cellular localization is shown in Figure 6A.

Finally, expression of NKAIN2 was investigated in the constitutional DNA of a cohort of 17 NB patients out of therapy by qPCR. As shown in Figure 6B, a significantly higher expression (P = 0.002) of *NKAIN2* was observed in the blood of NB patients as compared to age-matched healthy donors. Taken together these data suggest that high NKAIN2 expression by either the gene amplification or some other mechanisms may be relevant to NB pathogenesis.

## Discussion

Germline mutations in *ALK* and *PHOX2B* account for the majority of hereditary NBs. However, since these mutations are inherited and shared by unaffected parents, additional genetic events are thought to be required for tumor development.

To identify new candidate genes implicated in NB, we performed a comparison between somatic and germline DNA copy number changes in two NB-affected siblings carrying an *ALK* germline activating mutation (R1192P) This ALK mutation has been already reported in familial NB [14]. Array CGH analysis revealed a germline genomic gain in *NKAIN2* locus in one of the affected siblings, that was inherited from the parent who does not carry the *ALK* mutation. Surprisingly, *NKAIN2* was expressed at high levels also in the affected sibling that lacks the genomic gain at this locus, clearly suggesting mechanisms other than genomic gain involved in the gene regulation. We provided evidence that NKAIN2 play a role in NB development because its expression is directly related with the aggressiveness of human NB cell lines and tumor specimens and it is downregulated during ATRA differentiation. Herein, we distinguished two different NB cell phenotypes depending on the expression of NKAIN2: phenotype (a), neuroblastic cells that express high levels of NKAIN2; phenotype (b), neuroblastic cells with low NKAIN2 expression. The first phenotype was detected in the high-risk NB patients characterized by *MYCN* amplification, poor prognosis and therapy resistance. The second one features low-risk NB patients with low levels of MYCN associated with a relatively benign clinical profile, slow disease progression and therapy responsive. Furthermore, NKAIN2 was significantly expressed at higher levels in the peripheral blood of a cohort of NB patients as compared to age-matched healthy donors.

NKAIN2 belongs to a superfamily of transmembrane proteins interacting with β1 subunits of the Na/K-ATPase encoded by four genes, *NKAIN1*, *NKAIN2*, *NKAIN3* and *NKAIN4* [26]. *NKAIN* genes do not show any similarity with other known genes, although they share striking evolutionary conservation among species [26], [27]. Amino acid conservation of the first two domains suggests that this protein family might function within membrane bilayer. Some authors showed that a *Drosophila* mutant carrying a P-element insertion in the first exon of *Drosophila* ortholog *dNKAIN* displayed a temperature-sensitive paralysis [26]. The neuronal expression of NKAIN proteins, their membrane localization and the phenotype of NKAIN Drosophila mutants strongly suggest that these proteins are critical for neuronal function. In agreement with this hypothesis, two patients with severe neurological impairment have been found to express a truncated form of NKAIN2 by means of a constitutional chromosomal translocation [27], [28]. Because no other genes were disrupted, the clinical presentation of the two patients has been ascribed to *NKAIN2* truncation [27], [28]. As for NKAIN2 [26], mutations of Na/K-ATPase alpha subunits in *Drosophila* have been also found to cause neural dysfunction, leading to seizures and neurodegeneration, suggesting their fundamental role in maintaining normal neuronal functions [29].

Although NKAIN2 does not seem to have any specific enzymatic activity, it could be involved in some essential cellular pathway as a regulator. Previous studies have indicated that different NKAIN2 transcripts are expressed in fetal and adult brain suggesting that they may play a specific role at different stages [27]. In situ hybridization experiments confirmed the tissue-specific expression of NKAIN2 in the central and peripheral nervous system, with a distribution consistent with expression in neurons [26].

Herein, we provide evidence that NKAIN2 is downregulated in two NB cell lines by ATRA treatment, suggesting a role in neuronal differentiation. Retinoic acid exerts essential effects during embryonic development and differentiation of several cell types [30] and has been studied as human anti-cancer drug as it induces cell proliferation arrest, morphological differentiation and apoptosis in several cancer types, including NB [31], [32].

In summary, our data indicate that the *NKAIN2* might contribute to the elucidation of the complex mechanism of NB and its genetic components. Additional functional studies around this Na/K-ATPase interacting gene are required to further investigate its role in the NB susceptibility and to unveil the molecular basis of NB development and progression, hopefully providing diagnostic and therapeutic advances in this field.

## Materials and Methods

### NB cell lines

All human NB cell lines, obtained from the American Type Culture Collection, were characterized by morphology and HLA class I typing by PCR-SSP (Genovision). Cell lines were maintained in RPMI 1640 medium (HTLA-230, SK-N-BE(2), SK-N-BE(2)c, and IMR-32), Dulbecco's modified Eagle's medium (SH-SY5Y), or Minimun Essential Medium Eagle (SK-N-SH). All media were supplemented with 10% heat-inactivated fetal bovine serum (HyClone), 2 mM glutamine, 100 units/ml penicillin and 100 µg/ml streptomycin. Tet-21/N cell line was kindly provided by Dr M. Schwab (Johann Wolfgang Goethe-University, Frankfurt, Germany, [25]). All-trans retinoic acid (ATRA) and doxycycline, both from Sigma-Aldrich, were used at 10 µM and 10 ng/L, respectively.

### Ethics Statement

All tumor specimens and blood samples were obtained from NB patients diagnosed at the “Bambino Gesù” Children's Hospital, Rome (Italy), after obtaining written informed parental consent and approval by the Ethical Committee of the Institution. The clinical information of NB patients including patients' age at diagnosis, disease stage according to the International Neuroblastoma Staging System [18], *MYCN* oncogene copy number [33] and the International Neuroblastoma Pathology Classification [34] are listed in Table 2. They include 3 affected children of an Italian family of which two siblings and a first cousin, and 25 sporadic NB patients (Figure 1 and Table 2).

**Table 2:** Clinical-pathologic characteristics of NB patients.

Characteristics ategory	Number (%)
**Age** a	
<18	8 (29%)
≥18	20 (71%)
**INSS Stage**	
1, 2, 3, 4S	17 (61%)
4	11 (39%)
**MYCN**	
Amplified or gain	12 (43%)
Not Amplified	13 (46%)
Unknown	3 (11%)
**Histology**	
favorable	18 (64%)
unfavorable	10 (36%)

INSS, International Neuroblastoma Staging System; histology is based on International Neuroblastoma Pathology Classification (INPC).

a Age at diagnosis; months.

### Genomic DNA extraction

Genomic DNA was extracted from peripheral blood and tumor samples with High Pure PCR Template Preparation Kit (Roche) according to the producer's instructions. Concentration and purity of DNA samples were quantified by ND-1000 spectrophotometer (NanoDrop). The parental origin of the chromosome 15q14 deletion was assessed by studying a panel of short tandem repeats (STRs) using multiple primer pairs (available upon request) obtained from UniSTS database included in NCBI.

### DNA sequencing

Direct sequencing of the 3 exons of *PHOX2B* and of the exons 20 to 29 encoding the tyrosine kinase domain of *ALK* were performed using an Applied Biosystems 3500 Genetic Analyzer. Primers sequences and PCR conditions have been published elsewhere and are available [15], [35]. Mutant fragments were confirmed by bi-directional and repeated sequencing, establishing new PCR reactions. Sequence data were analyzed via Mutation Surveyor v. 3.30 software (SoftGenetics).

### Array-CGH and bioinformatic analysis

Array-CGH was carried out using a 60-mer oligonucleotide-based microarray platform that allows molecular profiling of genomic aberrations with an overall median probe spatial resolution of 13 Kb (180K) (Agilent Technologies Array-CGH Kits). Human genomic DNA was used as reference DNA. Aliquots of 2 µg of DNA from samples were double-digested with RsaI and AluI for 2 hours at 37°C. After heat inactivation of the enzymes at 65°C for 20 minutes, each digested sample was labelled by random priming (Agilent Technologies) for 2 hours using Cy5-dUTP for patient/parent DNAs and Cy3-dUTP for reference DNAs. Labelled products were column purified with Illustra CyScribe GFX purification kit (GE Healthcare). After probe denaturation and pre-annealing with 5-25 µg of Cot-1 DNA, hybridization was performed at 65°C with rotation for 24 hours. After washing steps, following the manufacturer's instructions, the array was analyzed using an Agilent scanner and Feature Extraction software v.10.5. A graphical overview of the results was obtained using DNA Analytics software v.4.0. The chromosome aberration regions were calculated by ADM2 algorithm with a moving average window of 50 Kb. The microarray data are available in the ArrayExpress database (www.ebi.ac.uk/arrayexpress) under accession number E-MEXP-3948.

To evaluate if the Copy Number Variations (CNVs) detected by array-CGH were polymorphic or potentially correlated with the clinical phenotype of the patients, bioinformatic analysis was carried out consulting the Database of Genomic Variants BioXRT [http://projects.tcag.ca/variation/]. Investigation of gene contents in the deleted segment was carried out by using UCSC database NCBI36/hg18 (http://www.genome.ucsc.edu).

### Quantitative mRNA expression

Total RNA was extracted using Trizol Reagent (Invitrogen). First-strand cDNA was synthesized from 1 µg using the SuperScript II First Strand cDNA synthesis kit (Invitrogen). Quantitative real-time PCR (qPCR) reactions were performed using an Applied Biosystems 7900-HT sequence detection system. Each sample was run in triplicate using pre-validated TaqMan gene expression assays from Applied Biosystems (Hs00902853_m1 for NKAIN2, Hs00900458_m1 for PCNX, Hs01036008_m1 for CASC4, Hs99999903_m1 for β-actin) under standard conditions. Relative gene expression was determined using the 2-Delta-Delta-Ct method [36], with β-actin as endogenous control.

### Antibodies, Immunoblotting and Immunohistochemistry

Whole-cell extracts obtained as previously described [24], were resolved on 8% SDS-PAGE and transferred onto Hybond-C paper (Amersham Bioscience). Filters were probed with anti-MYCN (sc-791, Santa Cruz Biotechnology), -NCAM1 (AB5032, Chemicon), or -b-actin (sc-1616, Santa Cruz Biotechnology) followed by incubation with peroxidase-coupled secondary Ab.

For immunohistochemical staining, tumor sections of paraffin-embedded tissue blocks were cut at 3 µm. Deparaffinization and antigen retrieval were performed with PT-link (Dako) in citrate buffer (pH 6.1) for 15 min at 98°C. Sections were incubated with the polyclonal rabbit anti-human NKAIN2 antibody (HPA045860, Sigma-Aldrich) for 45 min at room temperature followed by incubation with streptavidin alkaline phosphatase (Dako). Bound streptavidine was detected with Fast Red chromogene substrate (Dako) and levamisole in the reaction mixture for 10 min at room temperature. All samples were counterstained with haematoxylin. Sections of normal human placenta were used as positive control.

### Statistical Analysis

Statistical significance was assessed by Mann-Whitney test. A P value of <0.05 was considered to be statistically significant.

## Supporting Information

Figure S1 **Copy number profiles of chromosomes 6, 14 and 15 in the affected cousin.** The chromosome 6, 14 and 15 views (left) display the copy number profile of III-1 patient versus normal references. The gene views (right) magnify the selected amplification regions at 6q22, 14q24 and 15q15 indicating the log2 copy number ratios of individual oligos.
(TIF)

Figure S2 **All copy number alterations in the affected siblings are inherited from the father.** The chromosome views (left) display the copy number profile of constitutional DNA analysis from unaffected parents II-3 (A, C and E) and II-2 (B, D and F) of two affected siblings versus normal references. The gene views (right) magnify the selected amplification region at 6q22, 14q24 and 15q15 indicating the log2 copy number ratios of individual oligos.
(TIF)
