# Supplementary figures and images for: High-Resolution Array CGH Profiling Identifies Na/K Transporting ATPase Interacting 2 (NKAIN2) as a Predisposing Candidate Gene in Neuroblastoma
Source: PLoS One. 2013 Oct 25;8(10):e78481. doi: 10.1371/journal.pone.0078481 (PMC3808344; doi:10.1371/journal.pone.0078481)

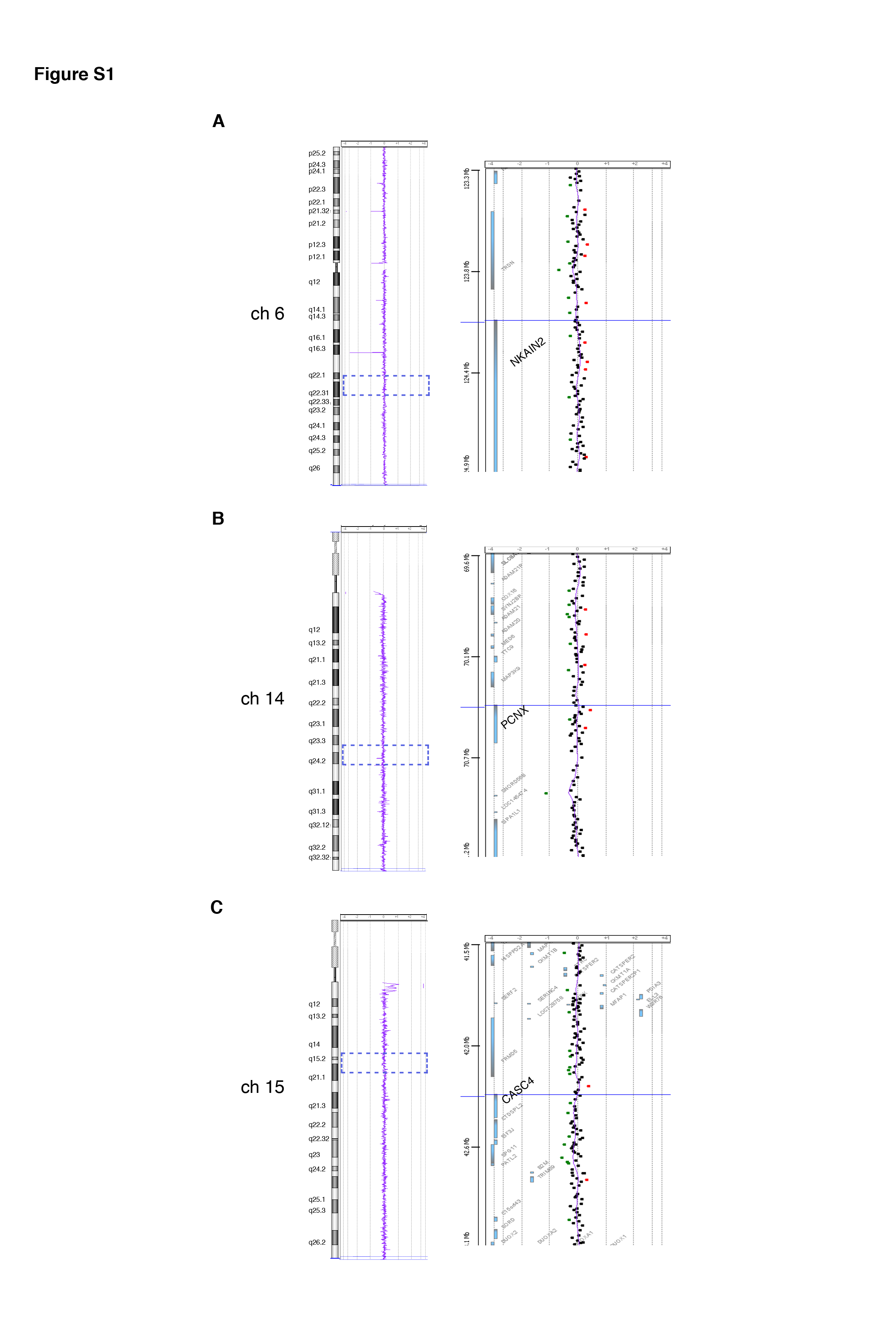

Supplement: Figure S1 — Copy number profiles of chromosomes 6, 14 and 15 in the affected cousin. The chromosome 6, 14 and 15 views (left) display the copy number profile of III-1 patient versus normal references. The gene views (right) magnify the selected amplification regions at 6q22, 14q24 and 15q15 indicating the log2 copy number ratios of individual oligos. (TIF) [file pone.0078481.s001.tif]

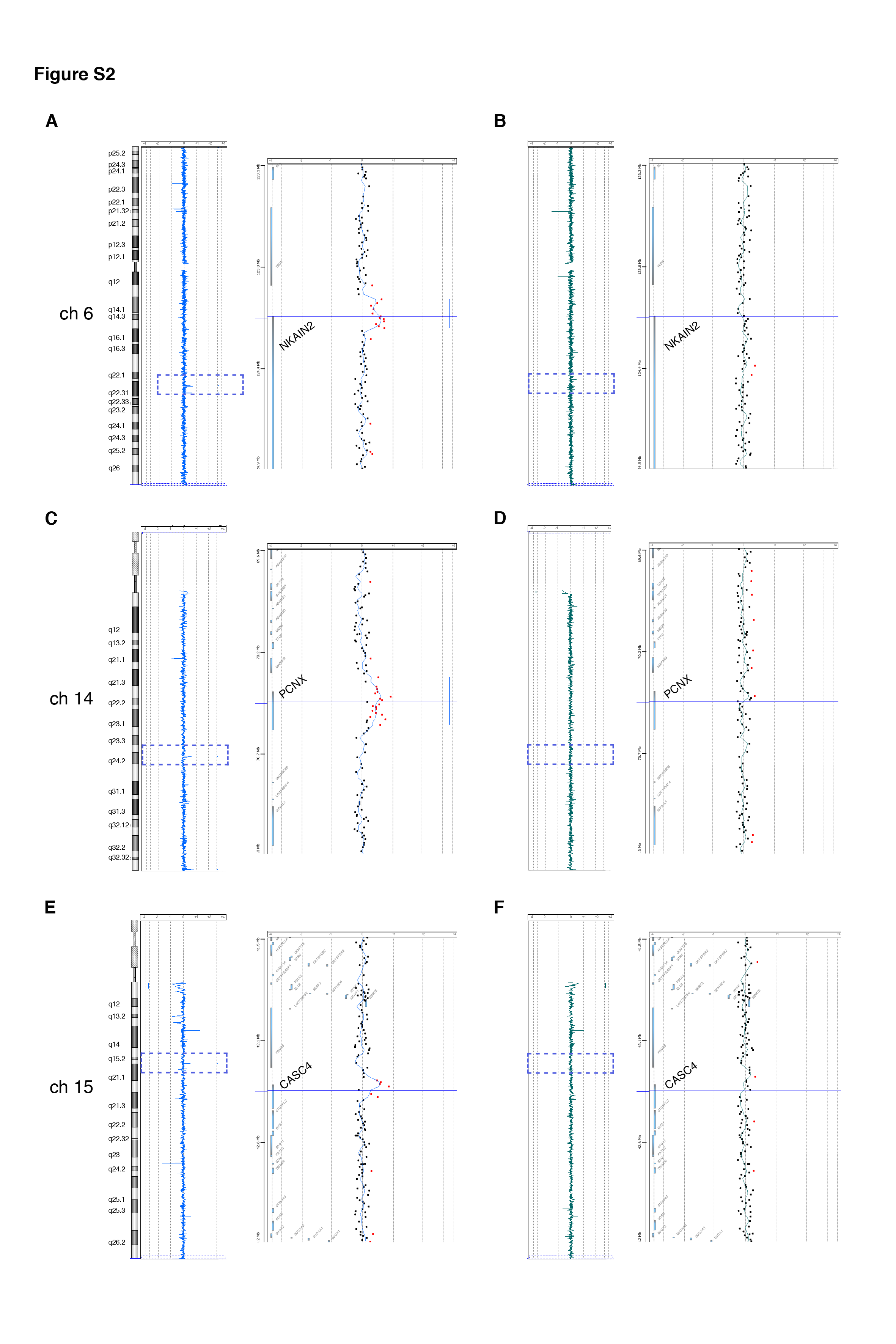

Supplement: Figure S2 — All copy number alterations in the affected siblings are inherited from the father. The chromosome views (left) display the copy number profile of constitutional DNA analysis from unaffected parents II-3 (A, C and E) and II-2 (B, D and F) of two affected siblings versus normal references. The gene views (right) magnify the selected amplification region at 6q22, 14q24 and 15q15 indicating the log2 copy number ratios of individual oligos. (TIF) [file pone.0078481.s002.tif]
